# A MIF haplotype is associated with the outcome of patients with severe sepsis: a case control study

**DOI:** 10.1186/1479-5876-7-100

**Published:** 2009-11-26

**Authors:** Lutz E Lehmann, Malte Book, Wolfgang Hartmann, Stefan U Weber, Jens-Christian Schewe, Sven Klaschik, Andreas Hoeft, Frank Stüber

**Affiliations:** 1 University Department of Anaesthesiology and Pain Therapy, Inselspital, CH-3010 Bern, Switzerland; 2 Department of Pathology, Bonn University, Sigmund-Freud-Str. 25, D-53105 Bonn, Germany; 3 Clinic and Policlinic for Anaesthesiology and Operative Intensive Care, Bonn University, Sigmund-Freud-Str. 25, D-53105 Bonn, Germany

## Abstract

**Background:**

Macrophage migration inhibitory factor (MIF) plays an important regulatory role in sepsis. In the promoter region a C/G single nucleotide polymorphism (SNP) at position -173 (rs755622) and a CATT_5-8 _microsatellite at position -794 are related to modified promoter activity. The purpose of the study was to analyze their association with the incidence and outcome of severe sepsis.

**Methods:**

Genotype distributions and allele frequencies in 169 patients with severe sepsis, 94 healthy blood donors and 183 postoperative patients without signs of infection or inflammation were analyzed by real time PCR and Sequence analysis. All included individuals were Caucasians.

**Results:**

Genotype distribution and allele frequencies of severe sepsis patients were comparable to both control groups. However, the genotype and allele frequencies of both polymorphisms were associated significantly with the outcome of severe sepsis. The highest risk of dying from severe sepsis was detectable in patients carrying a haplotype with the alleles -173 C and CATT_7 _(p = 0.0005, fisher exact test, RR = 1,806, CI: 1.337 to 2.439).

**Conclusion:**

The haplotype with the combination of the -173 C allele and the -794 CATT_7 _allele may not serve as a marker for susceptibility to sepsis, but may help identify septic patients at risk of dying.

## Background

Macrophage Migration Inhibitory Factor (MIF) is a cytokine widely expressed in both immune and non-immune cells playing an essential role in the pathophysiology of host immune and inflammatory responses [1,2]. After discovery for its name-giving activity of inhibiting the random migration of peritoneal macrophages [3], MIF was rediscovered as a hormone-like factor secreted by macrophages, anterior pituitary cells, and endothelial cells activating both macrophages and T-lymphocytes [4-7]. MIF was shown to be induced rather than suppressed by glucocorticoids and to have a capacity to override the anti-inflammatory and immunosuppressive effects of glucocorticoids [8]. Moreover, MIF was discovered to be involved in the regulation of cell membrane expression of TLR4, which mediates recognition of Gram-negative bacteria [9]. Also, MIF was shown to influence immuno-regulatory processes by indirectly affecting the transcriptional activity of nuclear transcription factor AP-1 [10]. The role of MIF in Gram-negative sepsis was reviewed by Roger and colleagues [11] and updated by Emonts and co workers [12]. Circulating concentrations of MIF were markedly elevated in children and adults who had severe sepsis or septic shock [12]. Circulating MIF levels are correlated with sepsis severity scores, presence of shock, disseminated intravascular coagulation, urine output, blood pH, and lactate and cytokine levels [12]. Moreover, high levels of circulating MIF are associated with a fatal outcome [12,13].

Consequently, MIF has been a relevant candidate gene for investigation in inflammatory disease and studies focusing on elucidation of MIF gene expression have been undertaken. Two functionally relevant promoter polymorphisms of the MIF gene have been described. A single nucleotide polymorphism (SNP) was identified in the untranslated 5' region of the MIF gene at position -173 consisting of a G to C transition (rs755622) [14]. Moreover, a tetranucleotide (CATT)_5-8 _repeat was found at position -794 [15]. Functional studies of both polymorphic sites have revealed altered MIF protein expression *in vitro*. Donn and co workers reported about the functional relevance of the -173 promoter SNP [16], whereas Baugh and co workers detected the -794 microsatellite and reported about the influence of the number of CATT repeats on the promoter activity [15]. In a large study Radstake and co workers reported the association of the -173 C allele and the -794 CATT_7 _microsatellite independently from each other with elevated circulating MIF levels in patients with rheumatoid arthritis [17]. Subsequently, higher MIF levels are correlated with more severe radiological joint damage [17].

The aim of this study was to evaluate the association of the MIF-173 G/C SNP and the MIF -794 CATT_5-8 _microsatellite with severe sepsis compared to healthy blood donors and patients with abdominal surgery but without signs of infection or inflammation. Secondly, an evaluation of the association of the MIF polymorphisms with the survival of severe sepsis patients was performed.

## Methods

The investigation was in compliance with the Helsinki declaration. After approval of the local ethics committee and written informed consent of the patient or a legal guardian had been obtained, 169 Caucasian patients with the diagnosis of severe sepsis according to the consensus conference [18] were included in the study. SOFA [19] scores were calculated, IL-6 and Procalcitonin (PCT) plasma levels were measured after the patients fulfilled criteria for severe sepsis. All patients were treated according to the surviving sepsis campaign guidelines [20]. Staff physicians were blinded of the patient's MIF genotype to avoid any bias in therapy. Two independent control groups were sampled, also: a) 183 Caucasian patients following major elective abdominal surgery without infectious or inflammatory complications and without post-operative ICU admission. b) 94 Caucasian healthy blood donors. All included individuals were Caucasians from Germany.

For genotyping of the MIF -173 promoter SNP 3.2 ml of whole blood were collected from each individual. DNA was prepared using the Flexi Gene DNA Kit (Qiagen, Hilden, Germany) according to the manufacturer's recommendations. Genotyping was done using a real-time PCR based system (LightCycler by Roche, Mannheim, Germany) with hybridization probes specific for the MIF -173 SNP. The PCR primer pair comprised of forward primer: 5' GGCTTCATCTCTGGAAGGGTAA, and reverse primer: 5' CAGCAACCGTCGCTAAGC. The sequence for the MIF -173 SNP specific hybridization anchor probe was: 5' GGCGGCTAGAAATCGGCCTGT. The sequence for the MIF -173 G/C SNP specific sensor probe was: 5' GCTCCAAGCTGTTCTCCAC. The anchor probe was phosphorylated at the 3' end and carried a LightCycler Red 640 (Roche) dye at the 5' end. The sensor probe was labeled by Fluorescein dye at the 5' end. Primers and probes were designed in cooperation with TIB-MOLBIOL (Berlin, Germany) and manufactured by this company. In brief the PCR was done using 45 cycles of 5 sec denaturation at 95°C, 8 sec annealing at 60°C and 8 sec of primer extension at 72°C. Subsequent melting curve analysis for determination of the MIF genotype was done with an initial 20 sec denaturation at 95°C, followed by an 60 sec annealing at 50°C and a final ramp to 85°C with continuous fluorescence acquisition at a transition rate of 0.1°C/sec. Additionally, individual samples representing the G/G, G/C or C/C genotypes as analyzed by real-time PCR were also genotyped by DNA sequencing to control for the accuracy of the real-time PCR method. All controlled samples had matching results between real-time PCR and DNA sequencing.

Genotyping of the MIF -794 microsatellite was performed analyzing a 130-142 bp PCR fragment covering a known CATT repeat in the 5'untranslated region of the gene. Primers used were MIF forward 5-TGTCCTCTTCCTGCTATGTC 3, and MIF reverse 5-CACTAATGGTAAACTCGGGG-3. The MIF reverse primer was 5-labeled with a fluorescent dye (5-FAM; MWG Biotech, Munich, Germany). The PCR program consisted of an initial denaturation for 5 min at 94°C followed by 34 cycles of 35 sec denaturation at 94°C, 40 sec annealing at 62°C, 40 sec extension at 72°C and a final extension step of 10 min at 72°C. The reaction products were analyzed on a semiautomated DNA sequencer (ABI 377) equipped with the Genescan software (ABI, Darmstadt, Germany). The coincidence of the -173 C allele and the -794 CATT_7 _microsatellite was described as an inferred haplotype.

Statistical analysis of genotype distribution and allele frequency was done by chi-square Test and Fischer's exact Test where applicable. The analysis of statistical differences of SOFA score and plasma levels (IL-6, PCT) was done by Mann-Whitney-U test. Bonferroni correction was applied for single marker analysis. Statistical significance was assumed at p < 0.05. Statistical power (1-β) was calculated using binominal power calculation. The power calculation for the -173 SNP and the -794 microsatellite based on investigations by Amoli and Baugh [15,21]. The relative risks of 2.1 and 1.56 were taken as a basis for effect sizes of a -173 and a -794 allele, respectively. Both relative risks were transferred from the mentioned publications [15,21]. The prevalence of severe sepsis was estimated to be 0.01. Using these preconditions the power of the presented results for the -173 G/C SNP and the -794 (CATT)_n _microsatellite was 98% and 87%, respectively. The linkage disequilibrium of the two loci was assessed with the open source application TASSEL2.1 http://www.maizegenetics.net. The association between the C and the CATT_7 _allele was assessed with a Fisher's exact test.

## Results

169 Patients with severe sepsis were studied (121 male and 48 female). 94 healthy blood donors and 183 patients after abdominal surgery without post-operative signs of infection or inflammation, without ICU admission and without case of death within the first postoperative 28 days served as two separate control groups. Furthermore, local wound infection rates reflected overall infection rates of this group of patients. The baseline characteristics of the both patient groups are outlined in Table 1. The mean age of healthy blood donors was 34 (18 to 56) and the female to male ratio was 37 to 57.

**Table 1:** Baseline characteristics of patients with severe sepsis and patients with abdominal surgery without signs of infection or inflammation (* unpaired t test, ** Fisher's exact test).

	Severe Sepsis (n = 169)	Abdominal surgery without infection/inflammation (n = 183)	p-value
Age (years)	58 (18 - 91)	56 (20 - 85)	0.16 *
Female:Male (n)	48 : 121	75 : 108	0.014 **
Source of infection			
Pulmonary (n)	67	n.a.	
Abdominal (n)	61	n.a.	
Other/unknown (n)	41	n.a.	
Mortality (%)	46.2	0	

Age is presented as mean and range of values. (n.a.: not applicable)

When dividing the severe sepsis patients in surviving (n = 91) and non-surviving patients (n = 78), mean SOFA score, as well as IL-6 plasma levels were significantly higher in non-surviving patients compared to survivors (Table 2). However, PCT plasma levels were comparable between the two groups (Table 2).

**Table 2:** Survivors and non-survivors of severe sepsis showed differences in SOFA score and IL-6 plasma level analyzed at the diagnosis of severe sepsis whereas PCT levels showed no significant differences (Mann-Whitney-U test).

	Survivors (n = 91)	Non-Survivors (n = 78)	p-value
SOFA Score	11 (7-20)	16 (14-22)	0.0256
IL-6 (pg/ml)	1677 (15-31860)	6497 (264-70361)	0.0098
PCT (ng/ml)	19.44 (9.45-46.49)	22.08 (2.03-246.1)	0.052

Data are presented as mean and range of values.

The allele CATT_8 _was neither detectable in the patients' group nor in either of the control groups. The genotype distribution and allele frequencies for the -173 SNP and the -794 microsatellite were comparable between the severe sepsis patients and the two control groups (p > 0.05, chi square test). There was no evidence of deviation from the Hardy-Weinberg equilibrium in the patient and control groups (p > 0.05, chi square test). Table 3 shows the genotype and allele distribution of both polymorphisms in the three groups. The carriage of the allele -173C was significantly associated with carriage of the -794 CATT_7 _microsatellite (Fisher's exact test, p < 0.0001, RR = 8.398, CI: 6.187 to 11.40). Moreover, both polymorphisms are in linkage disequilibrium (D' = 0.779).

**Table 3:** 173 and -794 genotype and allele distribution in the study population.

	-173							-794											Haplotype		
				
Study groups	Genotype				Allele			Genotype							Allele						
										
	G/G	G/C	C/C	p =	G	C	p =	5/5	5/6	5/7	6/6	6/7	7/7	p =	5	6	7	p =	-173 C and -794 CATT_7 _positive	-173 C and -794 CATT_7 _negative	p =
Healthy Controls (n = 94)	63	28	3	0.67	154	34	0.859	4	25	8	39	18	0	0.123	41	121	26	0.0451			
Abdominal surgery without infection or inflammation (n = 183)	123	54	6		300	66		7	55	7	75	35	4		76	240	50				
Severe sepsis (n = 169)	106	60	3		272	66		15	50	18	54	28	4		98	186	54				

Survivors sev. Sepsis (n = 91)	66	23	2	0.022	155	27	0.039	12	28	7	35	6	3	0.0016	59	104	19	0.0174	15	76	0.0005
Non-Survivors sev. Sepsis (n = 78)	40	37	1		117	39		3	22	11	19	22	1		39	82	35		32	46	

The 169 patients with severe sepsis are separated by surviving or non-surviving in the lower two rows. Genotype and allele distribution of healthy controls, patients with abdominal surgery, and patients with severe sepsis were tested by chi square test. Survivors and non-survivors of severe sepsis were also tested by chi square and Fischer's exact test where applicable, results were Bonferroni corrected. Incidence of the alleles -173 C **and **-794 CATT_7 _was associated with non-surviving severe sepsis (p = 0.0005, Fisher exact Test, RR = 1,806, CI: 1.337 to 2.439).

The genotype and allele frequencies of both polymorphisms were significantly different between survivors and non-survivors of severe sepsis (Table 3, -173 SNP genotype frequency: p = 0.0218, -173 SNP allele frequency: p = 0.0398, -794 microsatellite genotype frequency: p = 0.0016, -794 microsatellite allele frequency: p = 0.0174, chi-square test and Fischer's exact Test, respectively, all Bonferroni corrected).

Carrying the C allele of the -173 SNP resulted in a relative risk of 1.598 (CI: 1.165 to 2.193, p = 0.013, Fisher's exact test Bonferroni corrected, power: 1-β = 0.81) for a poor outcome of severe sepsis. Independently from the -173 SNP, the carriage of the -794 CATT_7 _allele showed a relative risk of 1.839 (CI: 1.360 to 2.488, p = 0.0006, Fisher's exact test Bonferroni corrected, power: 1-β = 0.96) for death due to severe sepsis compared to patients without allele CATT_7_. The concomitance of the -173 allele C and the -794 allele CATT_7 _as a haplotype was significantly associated with non-survival of severe sepsis (p = 0.0005, Fischer exact Test, RR = 1.806, CI: 1.337 to 2.439, power: 1-β = 0.91). Table 3 indicates the number of patients carrying the haplotype consisting of the -173 C and the -794 CATT_7 _allele. Accordingly, these patients display the -173 SNP C/C or G/C genotype in combination with the -794 microsatellite 5/7, 6/7 or 7/7 genotype, respectively.

Table 4 indicates the association of the carriage of the -173 C allele and the -794 CATT_7 _allele with fatal outcome in patients with severe sepsis which are grouped by the covariates "gender", "age", and "focus of infection". In the groups "female", "male", " ≤ 60 years old", and non-pulmonary and non-abdominal focus, carriage of -173 C allele and -794 CATT_7 _allele is associated with fatal outcome (table 4).

**Table 4:** Incidence of carrying the alleles -173 C and -794 CATT_7 _was associated with non-surviving severe sepsis in female and male patients, patients younger as 61 years old and patients with non-abdominal and non-pulmonary focus of sepsis.

Patients with severe sepsis sub groups	Sup group Mortality (%)	Association of allele carriage -173 C and -794 CATT_7 _and sub group status with fatal outcome		
		
		p	RR	CI
Gender				
Female (n = 48)	47.9	0.0135	2,182	1.251 to 3.805
Male (n = 121)	45.5	0.0267	1,635	1.126 to 2.373
				
Age				
≤ 60 y (n = 84)	41.7	0.0007	2,400	1.541 to 3.737
> 60 y (n = 85)	50.6	0.635	1,159	0.7499 to 1.790
				
Focus of infection				
Pulmonal (n = 67)	41.8	0,2312	1,514	0.8559 to 2.679
Abdominal (n = 61)	62.3	0,1734	1,337	0.9228 to 1.937
Other/unknown (n = 41)	29.3	0,0066	3,818	1.527 to 9.549

RR: Relative risk; CI: Confidence interval

## Discussion

The -794 MIF microsatellite is located in the promoter region and has functional relevance probably due to modified binding of nuclear transcription factors [22]. The relevance of the -794 microsatellite was assessed inconsistently with regard to analyzed cell types [15,22]. Just as well as the -794 microsatellite, the MIF -173 promoter SNP is of special interest because of its functional relevance. Donn and co workers detected increased promoter activity of the G allele compared to the C allele in an unstimulated lung epithelia cell line [16]. However, in the same work the authors reported about increased promoter activity of the C allele compared to the G allele in an also unstimulated T lymphoblast cell line [16]. The MIF plasma levels in -173 C carrying individuals were higher compared to non C carrying individuals [16]. Temple and co workers published about unstimulated and bacterial stimulated mononuclear cells in which allele -173 C occurrence resulted in decreased constitutive and inducible MIF mRNA levels [22]. This illustrates the complexity of the functional role of promoter polymorphisms in a comparatively simple *ex vivo *setting. Previous own results showed elevated MIF plasma levels in patients with severe sepsis as well as in patients with systemic inflammation compared to healthy controls [23].

Recent publications reported about the association of the -173 C allele with inflammatory diseases such as rheumatoid arthritis [17,24], inflammatory bowel diseases [25], and its clinical course [26]. The relevance of the C/C genotype was confirmed in Chinese patients with ulcerative colitis but not Crohn's disease [27]. The -794 microsatellite is also related to inflammatory diseases such as atopy [28], asthma [29], and rheumatoid arthritis [15]. Finally, the correlation of the -173 C allele and the -794 CATT_7 _allele as a haplotype with scleroderma [30], systemic lupus erythematosus [31] and with the susceptibility to psoriasis [32] was reported. A very recent publication reported about an association of -173 C allele carriage with lower 90 d mortality in a severe sepsis subgroup of patients with community-acquired pneumonia (CAP) which seems to be a contradiction to the presented results [33]. It has to be pointed out that Yende et al. reported a 90 day mortality rate of 27.2% in the severe sepsis subgroup which was lower compared to the 28 day mortality rate in the presented study (46.2%). This indicates that there might be elementary differences between both populations. Moreover, the independency of circulating MIF levels from the alleles is contradictory to previous reports [16]. Previous investigations in a Columbian population and Kenyan children showed the association (i) of the -173 C allele with tuberculosis and (ii) of the -173 C allele in combination with the -794 CATT_7 _microsatellite with severe malarial anemia, respectively [34,35]. These findings seem to be in line with our results reporting deleterious effects of these markers in infectious diseases. However, as discussed by Yende and co workers these differences may reflect the clinical heterogeneity in patients with infectious diseases. Finally, Yende and co workers recruited their individuals in the northeastern United States, whereas our patients and controls were from western Germany origin. Although both investigations included Caucasians genetic differences might contribute to the divergent results.

An approach to explain our findings might be the increased promoter activity combined with increased MIF plasma levels as reported by Donn and co workers for the -173 C allele. This might increase cardiomyocyte apoptosis as reported in an animal sepsis model [36]. In addition, MIF was associated with dysregulated pituitary-adrenal function in sepsis [12] and fatal outcome of severe sepsis [37].

The relevance of MIF polymorphisms in patients with sepsis was addressed only by association studies, so far. Gao and co workers reported about an association of the -173 SNP genotype C/C with the incidence of sepsis in African Americans but not in European Americans [38]. However, because of the limited sample size of -173 C/C individuals in Gao's investigation, this effect was assessed to be underpowered [38]. The number of individuals with the -173 C/C genotype in the present study is small as well and in the investigated Caucasian patients and control groups no significant association of the C/C genotype with the incidence of severe sepsis was detectable. It could be assumed, that different races might contribute to inconsistent associations due to different haplotype blocks. Gao and co workers reported about a haplotype in the European-descent American population which consists of the -173 promoter SNP, the promoter SNP rs9282783, the intron SNP rs2070777, the intergenic SNPs rs875643 and rs1007889 and the SNP rs2070767 which is located downstream of the 3' untranslated region [38]. Our results showed linkage disequilibrium between the two polymorphisms. This is in line with findings from Temple and co workers as well as Yende and co workers [22,33]. In addition, Temple and co workers reported the significant association of the -173 C allele with the -794 CATT_7 _allele [22], which was supported by our data. Generally, investigations analysing candidate genes in selected phenotypes can not exclude the detection of significant associations which are functionally inconsiderable but are in linkage disequilibrium with possibly undetected causative variants. However, there is some evidence for a functional impact of the investigated polymorphisms as pointed out above.

In the sepsis patient sub groups female and male patients as well as patients with age ≤60 years and in patients with non-pulmonary or non-abdominal focus the haplotype was associated with poor outcome whereas in older patients and in the groups defined by the focus of sepsis it was not. Especially in the younger patient group the effect of genetic predisposition might be stronger because of the fewer incidences of other confounders influencing the outcome. For example serious co-existing diseases like pulmonary, vascular or heart disease are well known as important comorbidities in the elderly. The association in the sub group with non-abdominal, non-pulmonary sepsis focus might be caused by higher influence of genetic predisposition in a group which is least ill reflected by the lowest mortality rate.

It is a general limitation of association studies that it is impossible to decide whether the investigated SNP has functional meaning or is in linkage disequilibrium with another, yet not identified variant which might be causal for functional implications. To date there are numerous reports indicating associations of SNPs with the incidence, course or outcome in sepsis or severe sepsis patients [39-43]. Although the SNPs are located within genes and some of them showed functional relevance in an *ex vivo *setting it is not for sure the case in a complex situation as *in vivo *blood stream infection.

Another serious point frequently addressed to association studies is the statistical power. The design of the present study included 169 patients with severe sepsis, 94 healthy controls and 183 surgical patients without infectious complications. 91 patients with severe sepsis survived and 78 patients died. Based on the allele, genotype and haplotype frequencies, on the fraction of non-surviving patients and on the genotype relative risks the statistical power of the significant associations were 81%, 96% and 91%, respectively. The relative risks of -173 and -794 alleles and genotypes reported by Baugh and Amoli [15,21] showed that the two polymorphisms have considerable high effect sizes for certain phenotypes. Our results confirmed these findings. The relative risks for poor outcome of the two polymorphisms and combination of both were 1.6, 1.84 and 1.80. This effect size was in line with the data published by Baugh and Amoli [15,21].

## Conclusion

The present study investigated for the first time the association of the MIF -173 promoter SNP and the MIF -794 CATT_5-8 _microsatellite with the incidence and outcome of severe sepsis by the comparison with two control groups. Our data indicate that neither alleles or genotypes of the -173 SNP variant nor of the -794 microsatellite were associated with the incidence of severe sepsis. However, at positions -173 and -794 alleles and genotypes were associated with survival of severe sepsis when analyzed separately as well as analyzed as a haplotype. Especially in the sub group of patients ≤60 years old and in patients with non-abdominal and non-pulmonary sepsis focus the association with poor outcome was pronounced. Of note, the common observation in both groups was the decreased mortality rate compared to the entire severe sepsis group. Therefore, in patients with severe sepsis and especially in younger patients with other than abdominal or pulmonary focus the MIF-173 G/C SNP and the -794 CATT_5-8 _microsatellite may not serve as markers for susceptibility to sepsis, but may well contribute to identify those septic patients at risk of dying.

## List of Abbreviations

AP-1: Activator protein-1; CI: Confidence interval; ICU: Intensive care unit; IL-6: Interleukin-6; MIF: Macrophage migration inhibitory factor; n.a.: not applicable; PCR: Polymerase chain reaction; PCT: Procalcitonin; RR: Relative risk; SNP: Single nucleotide polymorphism; SOFA: Sequential Organ Failure Assessment; TLR4: Toll like receptor 4.

## Competing interests

The authors declare that they have no competing interests.

## Authors' contributions

LEL planned the study, recruited patients, performed genotyping and drafted the manuscript. MB Performed statistical calculations, wrote the manuscript, contribute to patient inclusion and study design. WH contributed to study design, performed genotyping and drafted the manuscript. SUW contributed to patient inclusion, data analysis and drafted the manuscript. JCS contributed to patient inclusion and study design and drafted the manuscript. SK contributed to patient inclusion, genotyping and drafted the manuscript. AH contributed to study design, statistical calculations and drafted the manuscript. FS planned the study, supervised genotyping and statistical calculations, coordinated the study and drafted the manuscript. All authors read and approved the final manuscript

## References

[B1] Baugh JA Bucala R Macrophage migration inhibitory factor Crit Care Med 2002 30 S27 S35 10.1097/00003246-200201001-00004 11891404

[B2] Calandra T Roger T Macrophage migration inhibitory factor: a regulator of innate immunity Nat Rev Immunol 2003 3 791 800 10.1038/nri1200 14502271 PMC7097468

[B3] David JR Delayed hypersensitivity in vitro: its mediation by cell-free substances formed by lymphoid cell-antigen interaction Proc Natl Acad Sci USA 1966 56 72 77 10.1073/pnas.56.1.72 5229858 PMC285677

[B4] Bacher M Metz CN Calandra T Mayer K Chesney J Lohoff M Gemsa D Donnelly T Bucala R An essential regulatory role for macrophage migration inhibitory factor in T-cell activation Proc Natl Acad Sci USA 1996 93 7849 7854 10.1073/pnas.93.15.7849 8755565 PMC38837

[B5] Bernhagen J Calandra T Mitchell RA Martin SB Tracey KJ Voelter W Manogue KR Cerami A Bucala R MIF is a pituitary-derived cytokine that potentiates lethal endotoxaemia Nature 1993 365 756 759 10.1038/365756a0 8413654

[B6] Calandra T Bernhagen J Mitchell RA Bucala R The macrophage is an important and previously unrecognized source of macrophage migration inhibitory factor J Exp Med 1994 179 1895 1902 10.1084/jem.179.6.1895 8195715 PMC2191507

[B7] Nishihira J Koyama Y Mizue Y Identification of macrophage migration inhibitory factor (MIF) in human vascular endothelial cells and its induction by lipopolysaccharide Cytokine 1998 10 199 205 10.1006/cyto.1997.0276 9576065

[B8] Calandra T Bernhagen J Metz CN Spiegel LA Bacher M Donnelly T Cerami A Bucala R MIF as a glucocorticoid-induced modulator of cytokine production Nature 1995 377 68 71 10.1038/377068a0 7659164

[B9] Roger T David J Glauser MP Calandra T MIF regulates innate immune responses through modulation of Toll-like receptor 4 Nature 2001 414 920 924 10.1038/414920a 11780066

[B10] Kleemann R Hausser A Geiger G Mischke R Burger-Kentischer A Flieger O Johannes FJ Roger T Calandra T Kapurniotu A Grell M Finkelmeier D Brunner H Bernhagen J Intracellular action of the cytokine MIF to modulate AP-1 activity and the cell cycle through Jab1 Nature 2000 408 211 216 10.1038/35041591 11089976

[B11] Roger T Glauser MP Calandra T Macrophage migration inhibitory factor (MIF) modulates innate immune responses induced by endotoxin and Gram-negative bacteria J Endotoxin Res 2001 7 456 460 11753217

[B12] Emonts M Sweep FC Grebenchtchikov N Geurts-Moespot A Knaup M Chanson AL Erard V Renner P Hermans PW Hazelzet JA Calandra T Association between high levels of blood macrophage migration inhibitory factor, inappropriate adrenal response, and early death in patients with severe sepsis Clin Infect Dis 2007 44 1321 1328 10.1086/514344 17443469

[B13] Chuang CC Wang ST Chen WC Chen CC Hor LI Chuang AY Increases in serum macrophage migration inhibitory factor in patients with severe sepsis predict early mortality Shock 2007 27 503 506 10.1097/SHK.0b013e31802c024b 17438455

[B14] Donn RP Shelley E Ollier WE Thomson W A novel 5'-flanking region polymorphism of macrophage migration inhibitory factor is associated with systemic-onset juvenile idiopathic arthritis Arthritis Rheum 2001 44 1782 1785 10.1002/1529-0131(200108)44:8<1782::AID-ART314>3.0.CO;2-# 11508429

[B15] Baugh JA Chitnis S Donnelly SC Monteiro J Lin X Plant BJ Wolfe F Gregersen PK Bucala R A functional promoter polymorphism in the macrophage migration inhibitory factor (MIF) gene associated with disease severity in rheumatoid arthritis Genes Immun 2002 3 170 176 10.1038/sj.gene.6363867 12070782

[B16] Donn R Alourfi Z De BF Meazza C Zeggini E Lunt M Stevens A Shelley E Lamb R Ollier WE Thomson W Ray D Mutation screening of the macrophage migration inhibitory factor gene: positive association of a functional polymorphism of macrophage migration inhibitory factor with juvenile idiopathic arthritis Arthritis Rheum 2002 46 2402 2409 10.1002/art.10492 12355488

[B17] Radstake TR Sweep FC Welsing P Franke B Vermeulen SH Geurts-Moespot A Calandra T Donn R van Riel PL Correlation of rheumatoid arthritis severity with the genetic functional variants and circulating levels of macrophage migration inhibitory factor Arthritis Rheum 2005 52 3020 3029 10.1002/art.21285 16200611

[B18] American College of Chest Physicians/Society of Critical Care Medicine Consensus Conference: definitions for sepsis and organ failure and guidelines for the use of innovative therapies in sepsis Crit Care Med 1992 20 864 874 1597042

[B19] Vincent JL Moreno R Takala J Willatts S De MA Bruining H Reinhart CK Suter PM Thijs LG The SOFA (Sepsis-related Organ Failure Assessment) score to describe organ dysfunction/failure. On behalf of the Working Group on Sepsis-Related Problems of the European Society of Intensive Care Medicine Intensive Care Med 1996 22 707 710 10.1007/BF01709751 8844239

[B20] Dellinger RP Carlet JM Masur H Gerlach H Calandra T Cohen J Gea-Banacloche J Keh D Marshall JC Parker MM Ramsay G Zimmerman JL Vincent JL Levy MM Surviving Sepsis Campaign guidelines for management of severe sepsis and septic shock Intensive Care Med 2004 30 536 555 10.1007/s00134-004-2398-y 14997291

[B21] Amoli MM Donn RP Thomson W Hajeer AH Garcia-Porrua C Lueiro M Ollier WE Gonzalez-Gay MA Macrophage migration inhibitory factor gene polymorphism is associated with sarcoidosis in biopsy proven erythema nodosum J Rheumatol 2002 29 1671 1673 12180727

[B22] Temple SE Cheong KY Price P Waterer GW The microsatellite, macrophage migration inhibitory factor -794, may influence gene expression in human mononuclear cells stimulated with E. coli or S. pneumoniae Int J Immunogenet 2008 35 309 316 10.1111/j.1744-313X.2008.00781.x 18680514

[B23] Lehmann LE Novender U Schroeder S Pietsch T von ST Putensen C Hoeft A Stuber F Plasma levels of macrophage migration inhibitory factor are elevated in patients with severe sepsis Intensive Care Med 2001 27 1412 1415 10.1007/s001340000782 11511957

[B24] Martinez A Orozco G Varade J Sanchez LM Pascual D Balsa A Garcia A de la Concha EG Fernandez-Gutierrez B Martin J Urcelay E Macrophage migration inhibitory factor gene: influence on rheumatoid arthritis susceptibility Hum Immunol 2007 68 744 747 10.1016/j.humimm.2007.06.007 17869648

[B25] Oliver J Marquez A Gomez-Garcia M Martinez A Mendoza JL Vilchez JR Lopez-Nevot MA Pinero A de la Concha EG Nieto A Urcelay E Martin J Association of the macrophage migration inhibitory factor gene polymorphisms with inflammatory bowel disease Gut 2007 56 150 151 10.1136/gut.2006.107649 17172590 PMC1856656

[B26] Nohara H Okayama N Inoue N Koike Y Fujimura K Suehiro Y Hamanaka Y Higaki S Yanai H Yoshida T Hibi T Okita K Hinoda Y Association of the -173 G/C polymorphism of the macrophage migration inhibitory factor gene with ulcerative colitis J Gastroenterol 2004 39 242 246 10.1007/s00535-003-1284-7 15065001

[B27] Fei BY Lv HX Yang JM Ye ZY Association of MIF-173 gene polymorphism with inflammatory bowel disease in Chinese Han population Cytokine 2008 41 44 47 10.1016/j.cyto.2007.10.010 18054247

[B28] Hizawa N Yamaguchi E Takahashi D Nishihira J Nishimura M Functional polymorphisms in the promoter region of macrophage migration inhibitory factor and atopy Am J Respir Crit Care Med 2004 169 1014 1018 10.1164/rccm.200307-933OC 14962818

[B29] Mizue Y Ghani S Leng L McDonald C Kong P Baugh J Lane SJ Craft J Nishihira J Donnelly SC Zhu Z Bucala R Role for macrophage migration inhibitory factor in asthma Proc Natl Acad Sci USA 2005 102 14410 14415 10.1073/pnas.0507189102 16186482 PMC1242335

[B30] Wu SP Leng L Feng Z Liu N Zhao H McDonald C Lee A Arnett FC Gregersen PK Mayes MD Bucala R Macrophage migration inhibitory factor promoter polymorphisms and the clinical expression of scleroderma Arthritis Rheum 2006 54 3661 3669 10.1002/art.22179 17075815

[B31] Sanchez E Gomez LM Lopez-Nevot MA Gonzalez-Gay MA Sabio JM Ortego-Centeno N de RE Anaya JM Gonzalez-Escribano MF Koeleman BP Martin J Evidence of association of macrophage migration inhibitory factor gene polymorphisms with systemic lupus erythematosus Genes Immun 2006 7 433 436 10.1038/sj.gene.6364310 16724072

[B32] Donn RP Plant D Jury F Richards HL Worthington J Ray DW Griffiths CE Macrophage migration inhibitory factor gene polymorphism is associated with psoriasis J Invest Dermatol 2004 123 484 487 10.1111/j.0022-202X.2004.23314.x 15304087

[B33] Yende S Angus DC Kong L Kellum JA Weissfeld L Ferrell R Finegold D Carter M Leng L Peng ZY Bucala R The influence of macrophage migration inhibitory factor gene polymorphisms on outcome from community-acquired pneumonia FASEB J 2009 23 2403 2411 10.1096/fj.09-129445 19346297 PMC2717777

[B34] Gomez LM Sanchez E Ruiz-Narvaez EA Lopez-Nevot MA Anaya JM Martin J Macrophage migration inhibitory factor gene influences the risk of developing tuberculosis in northwestern Colombian population Tissue Antigens 2007 70 28 33 10.1111/j.1399-0039.2007.00843.x 17559578

[B35] Awandare GA Martinson JJ Were T Ouma C Davenport GC Ong'echa JM Wang W Leng L Ferrell RE Bucala R Perkins DJ MIF (macrophage migration inhibitory factor) promoter polymorphisms and susceptibility to severe malarial anemia J Infect Dis 2009 200 629 637 10.1086/600894 19591577 PMC3607439

[B36] Dhanantwari P Nadaraj S Kenessey A Chowdhury D Al-Abed Y Miller EJ Ojamaa K Macrophage migration inhibitory factor induces cardiomyocyte apoptosis Biochem Biophys Res Commun 2008 371 298 303 10.1016/j.bbrc.2008.04.070 18439909 PMC3104268

[B37] Bozza FA Gomes RN Japiassu AM Soares M Castro-Faria-Neto HC Bozza PT Bozza MT Macrophage migration inhibitory factor levels correlate with fatal outcome in sepsis Shock 2004 22 309 313 10.1097/01.shk.0000140305.01641.c8 15377884

[B38] Gao L Flores C Fan-Ma S Miller EJ Moitra J Moreno L Wadgaonkar R Simon B Brower R Sevransky J Tuder RM Maloney JP Moss M Shanholtz C Yates CR Meduri GU Ye SQ Barnes KC Garcia JG Macrophage migration inhibitory factor in acute lung injury: expression, biomarker, and associations Transl Res 2007 150 18 29 10.1016/j.trsl.2007.02.007 17585860 PMC1989118

[B39] Shalhub S Junker CE Imahara SD Mindrinos MN Dissanaike S O'Keefe GE Variation in the TLR4 gene influences the risk of organ failure and shock posttrauma: a cohort study J Trauma 2009 66 115 122 10.1097/TA.0b013e3181938d50 19131814 PMC2740632

[B40] Sipahi T Kuybulu A Ozturk A Akar N Protein Z G79A Polymorphism in Patients With Severe Sepsis Clin Appl Thromb Hemost 2009 http://cat.sagepub.com/cgi/content/abstract/1076029608330010v1 10.1177/1076029608330010 19124455

[B41] Russell JA Wellman H Walley KR Protein C rs2069912 C allele is associated with increased mortality from severe sepsis in North Americans of East Asian ancestry Hum Genet 2008 123 661 663 10.1007/s00439-008-0509-5 18496716

[B42] Henckaerts L Nielsen KR Steffensen R Van SK Mathieu C Giulietti A Wouters PJ Milants I Vanhorebeek I Langouche L Vermeire S Rutgeerts P Thiel S Wilmer A Hansen TK Van den BG Polymorphisms in innate immunity genes predispose to bacteremia and death in the medical intensive care unit Crit Care Med 2009 37 192 193 10.1097/CCM.0b013e31819263d8 19050632

[B43] Read RC Teare DM Pridmore AC Naylor SC Timms JM Kaczmarski EB Borrow R Wilson AG The tumor necrosis factor polymorphism TNF (-308) is associated with susceptibility to meningococcal sepsis, but not with lethality Crit Care Med 2009 37 1237 1243 10.1097/CCM.0b013e31819c39bc 19242354

